# Sexual Desire and Body Image. Gender Differences and Correlations before and during COVID-19 Lockdown

**DOI:** 10.3390/ijerph19074351

**Published:** 2022-04-05

**Authors:** Clemente Cedro, Carmela Mento, Maria Cristina Piccolo, Fiammetta Iannuzzo, Amelia Rizzo, Maria Rosaria Anna Muscatello, Gianluca Pandolfo

**Affiliations:** 1 Department of Biomedical, Dental and Morphological and Functional Imaging Sciences, University Hospital “G. Martino”, 98124 Messina, Italy; clemente.cedro@unime.it (C.C.); cmento@unime.it (C.M.); f.iannuzzo@libero.it (F.I.); maria.muscatello@unime.it (M.R.A.M.); gianluca.pandolfo@unime.it (G.P.); 2 Provincial Health Agency 5, 98123 Messina, Italy; cristina-piccolo@libero.it; 3 Psychiatry Unit, University Hospital “G. Martino”, 98124 Messina, Italy

**Keywords:** women, COVID-19, sexuality, body image

## Abstract

Recent literature has extensively examined sexual behavior during lockdown due to COVID-19. However, there are no recent studies that have considered the relationship between body image quality, sexual arousability, and sexual anxiety. The present study has two main objectives: (1) to examine gender differences in bodily and sexual experience; and (2) the comparison of bodily and sexual experience, before and during the COVID-19 lockdown. A total of 301 adult subjects (161 women and 140 men) aged between 16 and 73 years (Mean = 37.4; S.D. = 10.3) participated in the study. Data on biographical information were collected via an online panel. The Body Uneasiness Test (BUT) and the Sexual Arousability Inventory (SAI) were used for the assessment. Univariate ANOVA showed worse scores for women, compared with men, in terms of body image avoidance, depersonalization, overall severity of body image quality, sexual arousability, and sexual anxiety dimensions. When compared against time, only women showed significant correlations between the function of sexual arousal and all parameters concerning body image alteration. Interestingly, these correlations were weak and sporadic before lockdown, but strong and numerous during lockdown. This finding suggests that the impact of COVID-19 restrictions affected the female population more, with a profound repercussion on self-image and sexual and mental well-being.

## 1. Introduction

The COVID-19 pandemic has affected people’s sex lives. The lockdown period has radically changed interpersonal and inter-partner habits. Many factors such as the fear of contagion, the reduction of social relationships, higher chance of interpersonal conflicts, stress, lack of privacy, and the continued presence of children at home, have impacted sexual quality of life [1]. This has been evidenced by several multinational empirical data on the impact of restrictions and physical distancing on the quality of intimate life, although the nature of those changes does not appear homogeneous, and the issue is still unclear.

During this period of widespread movement and social contact restrictions, on average, the frequency of sexual behavior decreased. A recent study by Lehmiller et al. [2], on a English speaking sample mostly from the United States, 69% of whom were living with a partner and 31% were living alone, showed that many participants (43.5%) reported a decrease in sexual life quality; however, 42.8% reported that it remained the same, or even improved (13.6%). In particular, one in five participants reported expanding their sexual repertoire by incorporating new activities. Common additions included sexting, trying new sexual positions, and sharing sexual fantasies. Participants who made new additions were three times more likely to report improvements in their sex lives. Even in the face of drastic changes to daily life, many adults adapted their sex lives in more creative ways. 

Coombe et al. [3] also showed different sexual behaviors during lockdown in the Australian population. More specifically, authors found that most of the participants (53.5%) reported less sex during lockdown, compared with 2019. Thirty-five percent of participants were more likely to report sex with their spouse, and 45% were less likely to report sex with their partners or casual sex. 

Another study highlighted the decline in sexual arousal, specifically assessing the impact of COVID-19 measures on partner relationships and reproductive health in China. A sample of 3500 Chinese youth was recruited and interviewed about sexual desire, frequency of sexual intercourse, sexual satisfaction, etc. The questionnaire also collected demographic data (e.g., age, ethnicity, education, current financial situation, sexual orientation, relationship status, etc.). Due to the COVID-19 pandemic and related containment measures, 22% of participants (*n* = 212) reported a decrease in sexual desire; 41% (*n* = 396) reported a decrease in the frequency of sexual intercourse; 30% (*n* = 291) reported an increase in the frequency of masturbation; 20% (*n* = 192) reported a decrease in alcohol consumption before or during sexual activities; and 31% (*n* = 298) reported a deterioration in their relationship with their partner during the pandemic. These results show that many Chinese youths experienced significant changes during the lockdown, which affected their sexual and reproductive health [4].

Another study conducted in the United Kingdom examined a sample of 868 individuals during social self-isolation due to COVID-19, in which 39.9% of the sample reported engaging in at least one sexual activity once a week. Being male, a younger age, married, using alcohol frequently, and a greater number of days in self-isolation were associated with greater sexual activity, compared with women. Notably, 60.1% of the sample studied reported not being sexually active during social self-isolation [5]. 

The heterogeneity of results on sexual behavior during quarantine lead to the hypothesis that other psychological factors can mediate sexual well-being, satisfaction, and relationship quality, such as body image quality. Nevertheless, these studies reported sexual behavior alterations without considering one’s relationship with body image. Indeed, it is well known that interpersonal and sexual relationships are influenced by the relationship with the self and body [6,7]. Thus, it can be hypothesized that changes in sexual behavior during the lockdown may be tied to body image alteration, but this interesting hypothesis is still largely unexplored and deserves further analysis.

Body image has been defined as “the personal mental image of the shape, size and dimension of the body and the feelings we have about these characteristics and individual physical parts” [8,9]. Although it is experienced in the third person, body image cannot reach full objectivity, as it is influenced by personal attitudes, and various affective and behavioral components that ultimately modify the individual experience, thus impacting the self-esteem of the subject in particular [10]. Thus, it is the appraisals and emotions related to the experience of one’s body that ultimately determine an individual’s body satisfaction [11,12]. In the clinical setting, poor body satisfaction is linked to the onset and maintenance of eating or dysmorphic disorders [13], depression [14], anxiety [15], and substance use [16]. It has also been found in several independent studies that women generally report significantly lower levels of body satisfaction [17,18,19], resulting in a greater vulnerability to these issues.

The body image of each individual plays a key role in the sexual experience because it decisively contributes to sexual confidence. The possibility of feeling, or not feeling, satisfied with one’s own body determines a series of behaviors that inevitably reflect on sexuality [20]. The body is the vehicle of sexuality, it is mainly expressed with the body. A negative body image leads to low self-esteem, low body esteem, and dissatisfaction in one’s relationship with self-image and others [21]. Literature shows specific gender differences: on the one hand, women are more concerned about weight and body shape; on the other hand, men, in general, tend to be much more focused on the size of the genitals [22]. 

Scientific evidence suggests that there are gender differences in the perception of body image, in particular, greater body dissatisfaction and greater concerns about body shape have been found in women, rather than in men [23]. In addition, Dang et al. [24] found that sexual anxiety is mediated by factors such as sexual beliefs and attachment, and that this creates complex patterns, which take on different configurations in women and men.

The impact of the coronavirus has been undoubtedly crucial for people’s sex lives. It can be hypothesized that its effects will persist in the coming months or years, leading to unpredictable changes in relationships at all levels, not only in terms of affectivity but also in terms of sexual relationships. Psychological, social, and biological factors should be studied, especially regarding the possible increase in sexual desire disorders related to fear or anxiety. The possible reflections on the effects of lockdown in the relational and emotional sphere are indeed numerous, but unfortunately, there are still no systematic studies that have considered sexual behavior concerning body image quality during the pandemic.

Given these premises, the overall purpose of the present study is to investigate the possible effects of lockdown from COVID-19 on body experience, and the possible implications related to sexual functioning in the components of sexual desire, arousal, and anxiety. The specific objectives are (H1) to compare experiences before and during lockdown, taking into account the gender variable; and (H2) to verify the correlations between the different variables before and during, in the female sample.

## 2. Method

### 2.1. Procedure

The participants were invited to participate in the survey through mailing lists, instant messaging apps, and posts on Facebook groups, on a voluntary basis. Data were collected between June and October 2020 through Google’s telematics platform Forms ©. The research guaranteed anonymity, as requested by the ethical principles stated in the Declaration of Helsinki regarding subjects involved in research. The present survey did not involve any manipulation or treatment of data. 

Each participant, before completion, read and signed the informed consent form, in which the purpose of the research was explained: “The School of Psychiatry of Messina University (Italy), is investigating sexual excitability and anxiety associated with body image in relation to the lockdown period for COVID-19 Pandemic. We are interested in how body image-related discomfort, avoidant behaviors and compulsive control may affect people’s sexual life. You will be asked to answer short questionnaires. The survey will take about 15 min and respect anonymity and personal information, according to the current privacy law. The collected data will be used only for scientific research purposes. If you are under 18 years, you must ask your parents for consent. If you are taking the survey via cell phone, you will need to put your screen horizontally to view all response options. Thanks for your cooperation”.

Subsequently, the subjects were asked to fill out the biographical form containing general information on educational qualifications, occupation and marital status, as well as on romantic relationships and the two rating scales, in a single session lasting 15–30 min. The participants were asked to fill in the questionnaire, thinking retrospectively about their sexual and bodily experiences before the COVID-19 pandemic, and were then asked to give a score that compared them with the same experiences *during* the lockdown. The raw data were entered into an Excel spreadsheet for scoring, and were recoded and processed with the SPSS 25.0 Statistical Package for the Social Sciences (IBM-SPSS Inc., Chicago, IL, USA).

### 2.2. Instruments 

**Figure 1:**
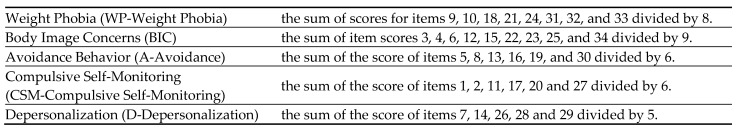
BUT factors.

The following two psychodiagnostic instruments were used to assess the variables considered. The Body Uneasiness Test (BUT) [25] is an assessment scale used for the study of body image and its pathologies. It is composed of 34 clinical items and 37 items related to specific experiences of body parts that consider the following dimensions: dissatisfaction and concern about one’s weight; concerns with body image; avoidance behaviors; compulsive control behaviors; feelings of detachment and estrangement from the body. Items are rated on a 6-point scale, from 0 (never) to 5 (always); higher scores indicate greater impairment. Several scores, indices, and factors can be obtained. Factor analysis isolated 5 factors (see Figure 1):

In addition to the total score, the Global Severity Index (GSI), or overall mean score, is calculated by summing the clinical scale scores and dividing by their number (34).

The second psychodiagnostic instrument used was the Sexual Arousability Inventory (SAI) [26]. This scale was created to assess the extent of arousal from different sexual experiences. The same instrument, in the so-called “expanded” version (Sexual Arousability Inventory-Expanded-SAI-E) used by us, can assess both the sexual arousability and anxiety that is aroused by the same sexual experiences. The scale, a self-assessment, is composed of 28 items that propose a series of situations of sexual involvement, in which the subject must say to what extent they are made excited or anxious. Each item is rated on a 7-point scale, from −1 (inhibition of arousal or relaxing effect) to 5 (constantly and extremely arousing, or constantly and extremely anxiety-provoking). The total score is obtained by summing the individual item scores (and subtracting the −1 scores). The higher the score, the greater the excitability or anxiety. In the present study, the SAI scale was replicated to provide four experimental conditions: (1) excitability before COVID-19; (2) sexual anxiety before COVID-19; (3) excitability during quarantine; and (4) sexual anxiety during quarantine. 

### 2.3. Participants

The interview was completed by a total of 301 participants, including 140 males and 161 females, ranging in age from 16 to 73 years (mean 37.4; S.D. 10.3); 2.6% (*n* = 8) of the participants stated that they suffered from a psychiatric pathology; 14.2% (*n* = 43) of the participants stated that they had undergone cosmetic surgery.

The sociodemographic variables examined are shown in Table 1.

## 3. Results

### 3.1. Statistical Analysis

Statistical analysis was performed using SPSS software version 25.0 for Windows. Descriptive statistics included a calculation of mean values (±SD) for all variables considered. Univariate ANOVA was used to assess changes in scores within the same sample before and during the lockdown. Spearman’s test was used for correlation analysis. After Bonferroni correction, *p* values less than 0.05 were considered statistically significant.

### 3.2. Gender Differences 

The first objective of the study was (H1) to compare the experience before and during the lockdown, taking into account the gender variable. The ANOVA test showed the significant impact of gender on body image and sexual related variables. Therefore, the groups of women and men were analyzed separately, with different results.

In the female group, both body variables, sexual arousal, and anxiety were significantly worse when comparing before and during the lockdown, with statistically significant differences. More specifically, a difference emerged in the following areas of bodily experience: avoidance behavior, depersonalization, and global severity index. On the other hand, weight phobia and compulsive self-image control are at the limits of significance (*p* = 0.05). Moreover, concerning sexual experience, there was a reduction in desire and an increase in anxiety (see Table 2).

In contrast, analysis of the male group related differences in body variables, and those related to sexual arousal and anxiety, before and during the lockdown, showed no quantitative or qualitative changes to any of the variables considered (See Table 3).

### 3.3. Correlations 

To further investigate the results that emerged, and (H2) to test the correlations between the different variables before and during lockdown, statistical correlation analyses were used in the female sample for both pre-lockdown and lockdown data.

Table 4 shows the correlations between body experience and sexual experience before lockdown in women. After Bonferroni correction, a *p* value of 0.006 was considered statistically significant. It should be noted that the subscales of the BUT show a strong internal correlation. Other significant correlations included:

- a negative correlation between weight phobia and age,
- a positive, but weak, correlation between compulsive self-image checks and sexual arousal,
- a positive correlation between depersonalization and sexual anxiety.

Table 5, on the other hand, shows the correlations between body experience and sexual experience in women during the lockdown. Highly significant correlations emerged between variables relating to body image alteration and sexual arousal and anxiety. The function of sexual arousal showed highly significant negative correlations with all parameters concerning body image alteration. In contrast, anxiety related to arousal factors showed highly significant positive correlations with all parameters concerning body image alteration.

## 4. Discussion

The present study investigated the relationship between body image quality and sexual excitability or anxiety before and during the COVID-19 pandemic. The most recent literature [1,2,4] reviewed, showed important alterations in sexual behavior that were independent from gender, geographical region, race, or sexual orientation. There is clear evidence that the lockdown has had an impact on the sexual behavior of millions of people. The theoretical framework on body image explains very well how sexuality is strongly related to body image: the body is a vehicle for the expression of sexuality, and consequently, alterations in self-image can hinder a healthy expression of excitability. We hypothesized that the two factors were related, although there are few studies linking body image and sexual arousability. This study fills a gap in a field of which we only have partial knowledge.

It is well known that body awareness is fundamental to identity, self-esteem, and self-value constructs. Body mediates identification and self-direction processes. Identity definition allows individuals to determine their priorities, to organize goals, and behaviors in a connected and coherent way, to direct actions and efforts effectively, to act consistently with personal values, to be resilient while maintaining levels of well-being and emotional satisfaction. A negative alteration of body image may be reflected in low quality of life and has often been found to underlie psychiatric disorders [27,28,29]. To date, the body image literature has focused on the relationship between an ideal of thinness and perfection, finding it linked to high rates of body dissatisfaction, as predictive factors for the development of eating disorders and depressive disorders, especially among women. 

The impact of the pandemic on sexuality has recently been the subject of several studies. The decrease in arousal, found in the present study, agrees with alterations found in other general population surveys conducted during the lockdown. However, the relationship between these alterations to sex life had not, thus far, been correlated with body image, taking into account gender differences. This makes the results original, but on the other hand, poorly comparable.

Our study showed that gender significantly affects body image perceptions, sexual arousability, and anxiety. In particular, the female sample showed a significant difference (i.e., a negative modification of the relationship with the body during the lockdown phase) compared with the previous period. This worsening phenomenon was also found in sexual arousal related functions and revealed an increase in sexual anxiety.

Interestingly, that sexual experiences were worsening was not valid for the male sample. This finding may underscore a greater predisposition of the female gender to respond to a peculiar situation, albeit a rare one, such as a pandemic lockdown, with bodily discomfort affecting sexual life. 

An interesting hypothesis is that the difference between men and women in the perception of their body image, and consequently in their quality of life, is influenced by both sociocultural and neurobiological factors [30,31]. From a neurobiological perspective, anxiety was found in subjects exposed to body image stimuli, and there was an increase in the activation of the amygdala as well as in the anterior cingulate cortex. Plausibly, this activation is the expression of mixed emotions, such as fear and anxiety, in response to negative body image related perceptions. Interestingly, this activation is not detected among males, and only characterized the women’s sample. This finding could lead to the hypothesis that the idea of thinness is rooted in a neurological circuit based on an emotional response among women, and this deep activation could be responsible for some aspects of eating and body image disorders [32].

From a socio-cultural perspective, there is a connection between social success and physical appearance, as well as between mental health and satisfaction with body image, especially for women. It has been found that women, compared to men, make more effort and spend more time to realize their ideal social image. In fact, they are more likely to resort to aesthetic surgery, nutritional and dietary counselling, and use more social comparison mechanisms. The most requested cosmetic surgery procedures by women are increase in breast size, followed by fat liposuction on buttocks, abdomen, and thighs, and this may suggest body image concerns [33]. 

In addition, gender differences are historically notable in terms of eating disorders such as anorexia nervosa and bulimia nervosa [34]. By adolescence, girls may become part of an all-female subculture in which topics of discussion and behaviors are associated with maintaining appearance, with dangerous psychopathological drifts. Recent studies show that adolescent females, given their vulnerable bodily self-esteem, are more likely to affiliate with blogs and groups that promote the development of eating disorders [35,36,37]. 

Increasingly, “sexiness” is also part of the ideal image of women. Women, and even adolescent girls, are encouraged to accept a view of sex that legitimizes their role as a sex object. In a study conducted at a college, the female sample reported a detailed list of characteristics that women consider to be fundamental to appear more sexy to men. Females included a range of activities, including shaving armpits, legs, arms, and genitals; wearing short skirts and bras that enhance the shape of the breast; caring and straightening hair; wearing a good perfume and bikini. In contrast, among the sample of young men belonging to the same college, it was found that the majority of men, to appear sexy to women, listed only two characteristics: wearing perfume and being clean [38,39].

Sexual hyper-investment in body image does not correspond to better bodily self-esteem nor sexual satisfaction, quite the contrary. It is important to emphasize that the data analyzed showed an absence of correlations between body experience and sexual arousal in women in the pre-lockdown phase, as opposed to the correlations that emerged in the lockdown period. This result suggests that the alteration of bodily experience in the lockdown phase has modified the experiences of sexual desire and arousal negative for women.

## 5. Limitations and Conclusions

Some limitations of the study need to be highlighted. The subjects responded to an online survey. Hence, the lack of control by the experimenter could not limit the effects of errors due to possible confounding factors in the subject’s environment. The effects of boredom, distraction, and social desirability were not estimated. 

The research design involved the dual administration of the same questionnaire. At first, subjects had to respond by thinking about their sexual experience before the lockdown, and then they had to respond again by thinking about their experience during the lockdown. This can generate several limitations, such as the halo effect. Another possible bias could be caused by responding retrospectively to a condition experienced in the past. The ideal condition would have been to detect the sexual experience at least six months earlier. However, no one could have predicted a worldwide pandemic. 

It should be also noted that conducting a techno-mediated survey reduces the target population to individuals who own an internet connection and digital devices such as personal computers, tablets, and smartphones. Moreover, most of the sample have high educational qualifications, which reduces the possibility of generalization to different social contexts or backgrounds. However, data collection, as outlined by the *APA in Opportunities and Challenges on Online Psychological Research* [40], was the only possible mode during a time of great restrictions, even for researchers. 

The lockdown had complex anthropological, social, and existential implications that are not yet sufficiently known. The pandemic factor, represented for the first time in history through continuous alarmist information from the mass media, has profoundly changed our perception towards the world and the others. Beyond the hypotheses that can be formulated, it appears evident from our preliminary data that women experience their relationships to their bodies and sexualities with greater discomfort.

Although the understanding of this gender difference deserves in-depth studies that necessitate being targeted and interdisciplinary, a contribution that this work is that it can give greater attention to the level of support and psychological assistance that women need at this time in history, and that is expected to continue for a long time.

Government initiatives related to emergency management have included psychological care planning focused on COVID-19 positive patients, and health care workers undergoing severe stress and traumatic experiences. However, the need for the psychological support of the general population has not been fully realized. 

The results of this study highlight that psychological support represents a central, albeit underestimated, health factor. Particular attention should be directed towards the female population, since some aspects of mental suffering seem to be particularly related to gender.

Psychological interventions should be targeted and specific considering that we are facing a completely new phenomenon that could undermine the psychological balance in the general population, laying the groundwork for future psychopathological expressions that are not predictable.

## Figures and Tables

**Table 1:** Sociodemographic characteristics of the sample.

**Sentimental Status**	No relationship	11%
	In a stable relationship	66.4%
	In a complicated relationship	9.6%
	In an open relationship	4.7%
	Occasional relationship	8.3%
Profession	Student	8.3%
	Employee	44.9%
	Self-employed	35.5%
	Unemployed	5.6%
	Other	5.6%
Education	Primary school certificate	3.1%
	Secondary school certificate	6.9%
	High School Diploma	25.9%
	Degree	64.1%
Income range	From EUR 0 to 36.152	57.5%
	From EUR 36.153 to 70.000	29.6%
	From EUR 70.001 to 100.000	10.2%
	Over EUR 100.000	2.7%

**Table 2:** Comparison of body variables and sexual arousal and anxiety before and during the lockdown in Women.

Women’s Scores (*n* = 161)		Mean	Std. Deviation	F	Sig
Weight Phobia	before	1.9	1.2	3.661	0.057
	during	2.2	1.7		
Body Image Concern	before	1.7	1.2	1.668	0.197
	during	1.9	1.6		
Avoidance	before	8.7	1.0	4.885	0.028
	during	1.2	1.6		
Compulsive self-monitoring	before	1.4	1.0	3.669	0.056
	during	1.7	1.5		
Depersonalization	before	0.8	1.1	5.702	0.018
	during	1.2	1.7		
Global Severity Index	before	1.4	1.0	3.937	0.048
	during	1.7	1.5		
Sexual Arousability	before	113.24	32.43	13.984	0.000
	during	97.50	42.43		
Sexual Anxiety	before	15.45	33.75	11.924	0.001
	during	31.87	50.01		

**Table 3:** Comparison of body variables and sexual arousal and anxiety before and during the lockdown in men.

Men’s Scores (*n* = 140)		Mean	Std. Deviation	F	Sig
Weight Phobia	before	1.2	1.1	0.142	0.707
	during	1.3	1.5		
Body Image Concern	before	1.1	1.1	0.006	0.938
	during	1.1	1.5		
Avoidance	before	0.7	1.1	0.471	0.493
	during	0.8	1.4		
Compulsive self-monitoring	before	1.0	0.9	0.314	0.575
	during	1.1	1.4		
Depersonalization	before	0.7	1.1	1.347	0.247
	during	0.9	1.5		
Global Severity Index	before	1.0	1.0	0.223	0.637
	during	1.1	1.4		
Sexual Arousability	before	125.53	29.82	0.296	0.587
	during	123.16	42.10		
Sexual Anxiety	before	35.86	60.39	1.264	0.262
	during	43.82	58.10		

**Table 4:** Correlations between body image dimensions, arousal, and sexual anxiety in women before lockdown.

	Weight Phobia	BIC	AV	CSM	DEP	GSI	Sex Arous.	Sex Anx
BIC-Body Image Concern	0.892 **							
AV-Avoidance	0.718 **	0.812 **						
CSM-Compulsive self-monitoring	0.803 **	0.780 **	0.733 **					
DEP-Depersonalization	0.676 **	0.752 **	0.882 **	0.737 **				
GSI-Global Severity Index	0.928 **	0.955 **	0.892 **	0.883 **	0.861 **			
Sexual Arousability	−0.039	0.033	0.104	0.161 *	0.104	0.061		
Sexual Anxiety	0.021	0.087	0.153	0.131	0.167 *	0.108	0.131	
Age	−0.171 *	−0.151	−0.065	−0.111	−0.081	−0.139	−0.027	0.000

Legend: Correlation is significant at ** 0.01 level (2-tailed) * 0.05 level (2-tailed).

**Table 5:** Correlations between body image size and sexual arousal and anxiety in women during lockdown.

	Weight Phobia	BIC	AV	CSM	DEP	GSI	Sex Arous.	Sex Anx.
BIC-Body Image Concern	0.937 **							
AV-Avoidance	0.840 **	0.892 **						
CSM-Compulsive self-monitoring	0.880 **	0.885 **	0.845 **					
DEP-Depersonalization	0.825 **	0.895 **	0.946 **	0.866 **				
GSI-Global Severity Index	0.953 **	0.977 **	0.942 **	0.937 **	0.942 **			
Sexual Arousability	−0.259 **	−0.273 **	−0.397 **	−0.280 **	−0.403 **	−0.327 **		
Sexual Anxiety	0.578 **	0.626 **	0.668 **	0.600 **	0.688 **	0.657 **	−0.280 **	
Age	−0.193 **	−0.163 *	−0.120	−0.128	−0.088	−0.153	−0.050	−0.114

Legend: Correlation is significant at ** 0.01 level (2-tailed) * 0.05 level (2-tailed).

## Data Availability

Not available.
